# Co-creating Humanistic AI AgeTech to Support Dynamic Care Ecosystems: A Preliminary Guiding Model

**DOI:** 10.1093/geront/gnae093

**Published:** 2024-08-02

**Authors:** Amy S Hwang, Thomas Tannou, Jarshini Nanthakumar, Wendy Cao, Charlene H Chu, Ceren Zeytinoglu Atici, Kerseri Scane, Amanda Yu, Winnie Tsang, Jennifer Chan, Paul Lea, Zelda Harris, Rosalie H Wang

**Affiliations:** Department of Occupational Science and Occupational Therapy, University of Toronto, Toronto, Ontario, Canada; Centre de Recherche, Institut Universitaire de Gériatrie de Montréal, CIUSSS Centre-Sud de l’Ile de Montréal, Montréal, Québec, Canada; Centre de Recherche, Institut Universitaire de Gériatrie de Montréal, CIUSSS Centre-Sud de l’Ile de Montréal, Montréal, Québec, Canada; Department of Occupational Science and Occupational Therapy, University of Toronto, Toronto, Ontario, Canada; Independent Research Consultant, Toronto, Ontario, Canada; Lawrence S. Bloomberg Faculty of Nursing, University of Toronto, Toronto, Ontario, Canada; Başlangıç Noktası (Be Node), Turkish Informatics Foundation, Istanbul, Turkey; Independent Research Consultant, Toronto, Ontario, Canada; Independent Research Consultant, Toronto, Ontario, Canada; Good Works Collective Inc., Toronto, Ontario, Canada; Independent Research Consultant, Toronto, Ontario, Canada; Independent Research Consultant, Toronto, Ontario, Canada; Independent Research Consultant, Toronto, Ontario, Canada; Department of Occupational Science and Occupational Therapy, University of Toronto, Toronto, Ontario, Canada

**Keywords:** Caregiving, Digital ageism, Ethics, Robot, Smart home

## Abstract

As society rapidly digitizes, successful aging necessitates using technology for health and social care and social engagement. Technologies aimed to support older adults (e.g., smart homes, assistive robots, wheelchairs) are increasingly applying artificial intelligence (AI), and thereby creating ethical challenges to technology development and use. The international debate on AI ethics focuses on implications to society (e.g., bias, equity) and to individuals (e.g., privacy, consent). The relational nature of care, however, warrants a humanistic lens to examine how “AI AgeTech” will shape, and be shaped by, social networks or care ecosystems in terms of their care actors (i.e., older adults, care partners, service providers); inter-actor relations (e.g., care decision making) and relationships (e.g., social, professional); and evolving care arrangements. For instance, if an older adult’s reduced functioning leads actors to renegotiate their risk tolerances and care routines, smart homes or robots become more than tools that actors configure; they become semiautonomous actors, in themselves, with the potential to influence functioning and interpersonal relationships. As an experientially diverse, transdisciplinary working group of older adults, care partners, researchers, clinicians, and entrepreneurs, we co-constructed intersectional care experiences, to guide technology research, development, and use. Our synthesis contributes a preliminary guiding model for AI AgeTech innovation that delineates humanistic attributes, values, and design orientations, and captures the ethical, sociological, and technological nuances of dynamic care ecosystems. Our visual probes and recommended tools and techniques offer researchers, developers/innovators, and care actors concrete ways of using this model to promote successful aging in AI-enabled futures.

## Humanistic AI AgeTech: A New Dimension to Ethical and Responsible AI

With society rapidly aging and digitizing, and fewer available caregivers, individuals, and systems are seeking technological innovation to support older adults with care needs (Schulz et al., 2020). Products and services are increasingly applying artificial intelligence (AI) algorithms that can learn, predict, and (semi-) autonomously respond to human behavior, offering functions to support older adults and care partners (“AI AgeTech,” referred hereafter). Smart homes can control environmental features (e.g., temperature), monitor safety events (e.g., detect falls), and facilitate social interactions (e.g., video calls)—all intended to help older adults age-in-place (Facchinetti et al., 2023). Assistive robots may reduce isolation and loneliness, improve sociability, and stimulate cognitive function (Abdi et al., 2018). Intelligent wheelchairs may facilitate users’ independent mobility, overcoming cognitive barriers to driving (Viswanathan et al., 2017). Overall, these innovations may preserve independence, functioning, and dignity for older adults while supporting care partners.

Amidst urgent calls for ethical AI across sectors (Bengio, 2023; The White House, 2023), many ethical debates have centered on personal privacy and data security; informed consent (i.e., possessing adequate information and transparency to make decisions about one’s participation); autonomy (i.e., the right to make decisions concerning oneself); discrimination (i.e., unjust treatment based on prejudice); and bias (i.e., prejudice in favor of one group over another; Wang et al., 2023). Avoiding “digital ageism” necessitates mitigating the development and implementation of AI-based technologies that produce or perpetuate age-related prejudice (Chu et al., 2021, 2023). Supporting technology adoption by persons with dementia requires consideration of “ethical adoption” (Robillard et al., 2018) and the biomedical ethical principles of autonomy, beneficence (i.e., balancing benefits/risks), nonmaleficence (i.e., avoiding causing harm), and justice (i.e., distributing costs, risks, and benefits fairly across individuals; Beauchamp & Childress, 2019). Ethical adoption (Robillard et al., 2018) is founded on the pillars of inclusive participatory design; (technology) adoption modeling; ethical standards assessment; and education/training (i.e., leveraging familiarity, minimizing training needs, optimizing technological support). Additional ethical concepts warranting reflection for smart homes and assistive robots include the potential for reduced human contact; felt objectification (e.g., promoting care efficiency over human empathy) or loss of control or liberty; and infantilization or deception that a robot is capable of emotional connection (Sharkey & Sharkey, 2012; Zhu et al., 2022). Together, these ethical discussions have largely focused on implications to individuals and society, and primarily consider interactions between older adults and “the AI” (hereafter, referring to an AI AgeTech product or service).

**Figure 1.**
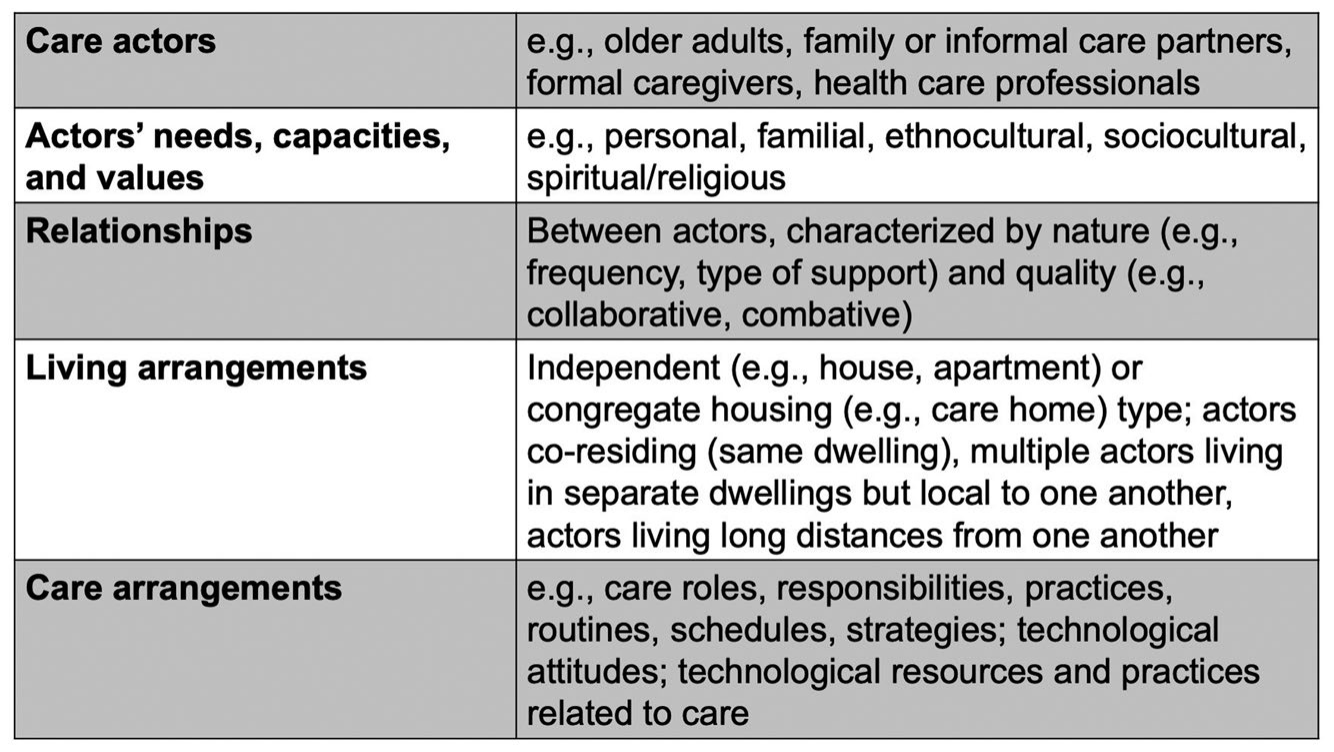
Attributes of a care ecosystem. Adapted from Atlas of Care (2019) and Hwang (2019).

Care, however, is relational in nature (Dupuis et al. 2016; Hwang, 2019; Nolan et al., 2004) and requires a framework, beyond the realm of applied ethics, to guide how AI may contribute to caring for and with older adults. Thus, we adopt a humanistic lens that focuses on the complexity of interpersonal relationships (Leibing, 2019) and the plurality of values (Tasioulas, 2022) that constitute caring. We employ the concept of *care ecosystems* (Figure 1)—dynamic networks comprising multiple care actors, relationships, and various forms of support between actors (Atlas of Care, 2019; Hwang, 2019). We then integrate a perspective that technology adoption is also a relational experience (Carroll, 2004; Hwang, 2019; McCarthy & Wright, 2004). When technologies are introduced into a care ecosystem, its actors, their behaviors, and their relationships may transform as they mutually adapt to and while “appropriating” the technology (Hwang et al., 2020). The AI may not only facilitate or mediate these changes; the AI itself may become an actor (Guzman & Lewis, 2020; Rattay et al., 2023), sensing, learning, and responding (semi-) autonomously, and potentially developing unique relationships with the human actors. To promote the *humanistic* creation of AI AgeTech, we propose a preliminary guiding model based on a transdisciplinary synthesis of humanistic attributes, values, and design orientations, and illustrative tools and techniques for future innovation.

## Mobilizing Transdisciplinary and Intersectional Collaboration

**Figure 2.**
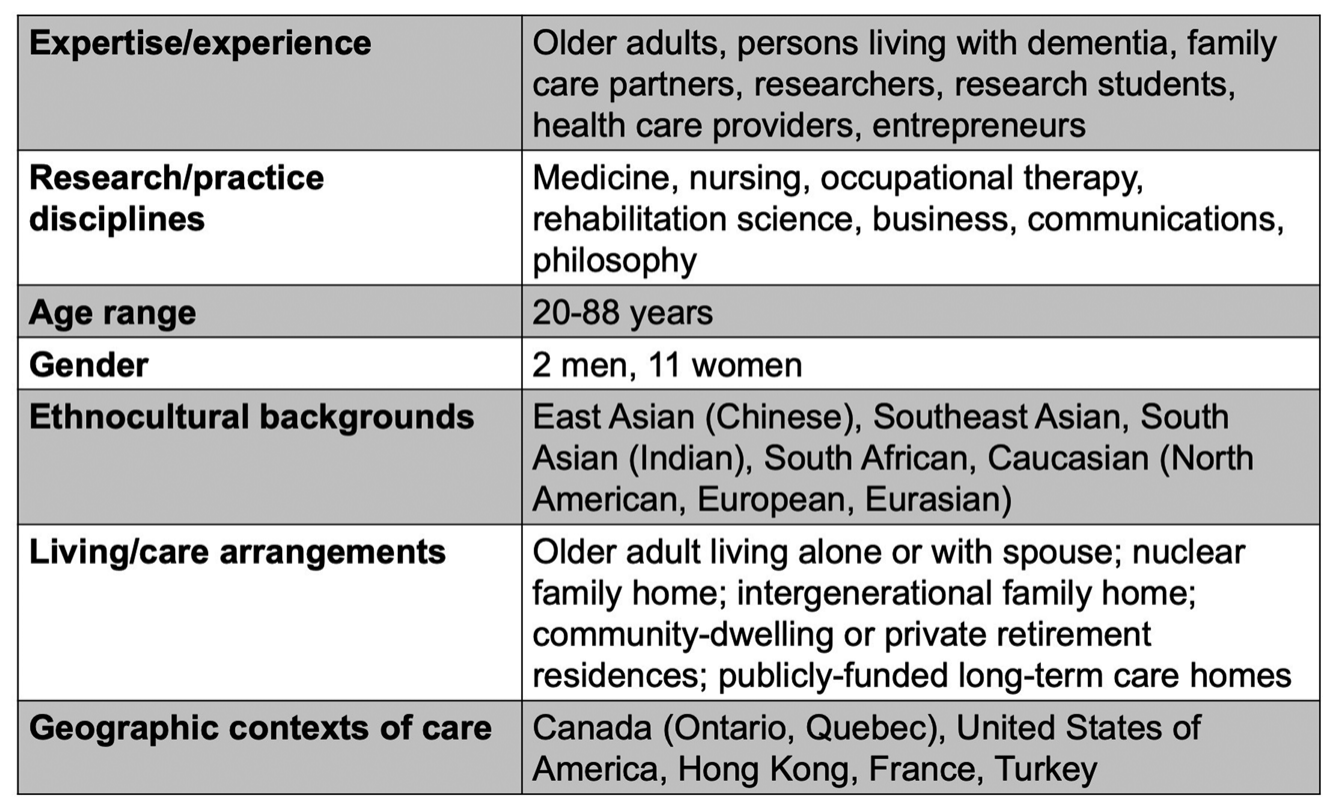
Intersectional experiences of Working Group (WG). Self-identified information from working group members. Members may identify with multiple categories.

We formed a diverse, transdisciplinary working group (WG; *N* = 13; all coauthors) where members contributed their diverse and intersectional care experiences (Figure 2).

**Figure 3.**
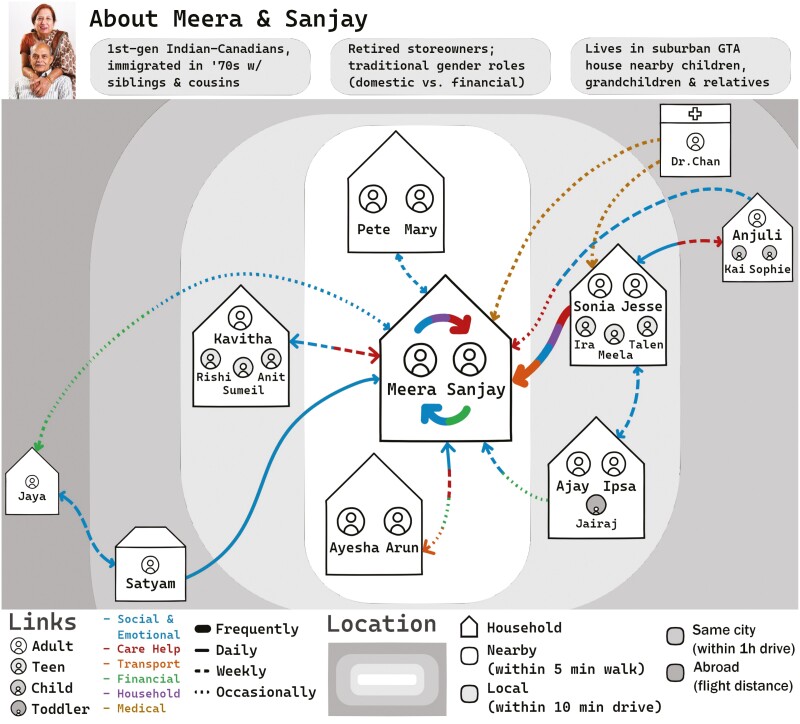
Example of co-created persona and care map. Our care map of persona, Meera and Sanjay, co-created by our working group to instantiate a dynamic care ecosystem encompassing different actors and interdependent relationships that vary in nature and frequency of support. Personas were developed with guidance from (Pruitt & Adlin, 2010) and the care map was developed with guidance from (Atlas of Care, 2019). See full personas in Supplementary Figure 1. GTA = Greater Toronto Area.

We approached our WG engagement from different theoretical orientations. We adopted a humanistic lens and relational caring perspective that recognizes different actors’ needs, values, and perspectives; the reciprocal and interdependent nature of the relationships between actors; and the dynamic nature of care decisions and interactions (Dupuis et al., 2016; Hwang, 2019; Nolan et al., 2004). We applied the concept of care ecosystems (Figures 1 and 3) that change over time and circumstance and considered different scenarios that may create ethical dilemmas between actors (Holt, 2020). We assumed technology to be more than a tool, but rather a relational and felt experience (McCarthy & Wright, 2004), through which actors shape, and are shaped, by their interactions (Carroll, 2004). We considered AI AgeTech to possess a degree of autonomy (e.g., independent decision making) and intelligence (e.g., ability to learn), with the potential to become a care actor (Guzman & Lewis, 2020; Rattay et al., 2023), and interact with human actors as embodied and/or pervasive (i.e., environmentally embedded) forms.

We applied co-creation as a participatory mindset and method, engaging in generative sessions using tools and techniques to generate tacit and latent knowledge, evoke feelings, and explore a future design space (Sanders & Stappers, 2013). One researcher (A.S. Hwang) facilitated three generative sessions over 2.5 months (total 5 hr via Zoom, 7 hr in-person). To foster a common vocabulary and stimulate co-creation, A.S. Hwang and R.H. Wang developed and presented visual probes (Sanders & Stappers, 2013) to introduce the care ecosystem concept (Figure 3) and AI AgeTech (i.e., smart homes, assistive robots, intelligent wheelchairs).

**Figure 4.**
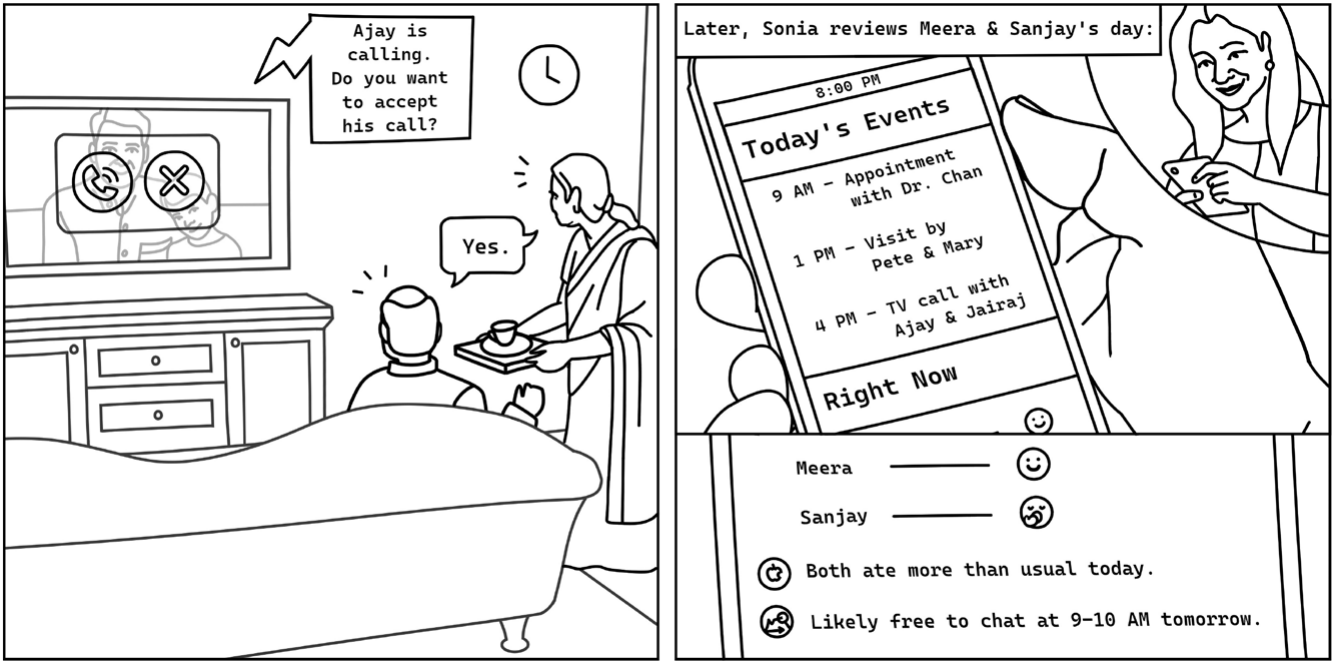
Example of a co-created AI AgeTech scenario. A smart home scenario co-created by our working group for Meera and Sanjay, guiding AI AgeTech design. The system, on the left, shows an incoming video call, automatically detecting their availability in the living room to solve the problem of “phone tag.” On the right, their daughter (primary care partner) views a summary of their activities, moods, and health behaviors, with recommendations for timing social interactions accordingly. This assists in coordinated caregiving activities and social support between Meera’s and Sanjay’s care partners. A report (bottom right) indicates their improved well-being through social contact, providing their daughter both positive feedback and guidance for efficiently planning her support/visits.

**Figure 5.**
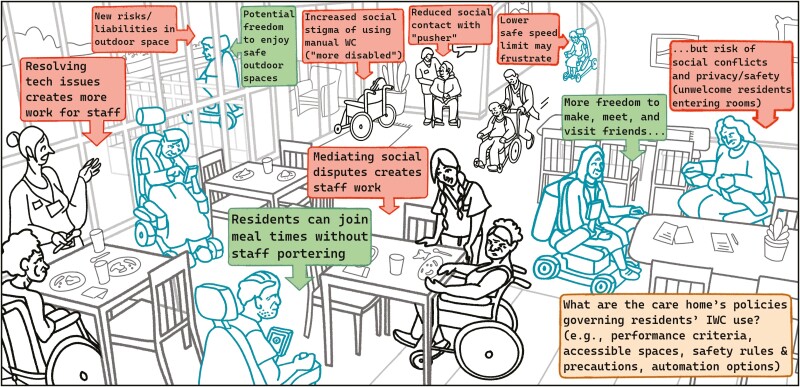
Hypothetical intelligent wheelchair scenarios in care home settings. Scenarios envisage intelligent wheelchairs in care home settings, based on qualitative insights by (Wang et al., 2011), and were employed as visual probes to stimulate working group discussion. Over multiple co-creation sessions, our working group annotated the scenario illustrations with potentially positive and negative consequences that intelligent wheelchairs may create in care home settings. IWC = intelligent wheelchair.

We co-created personas (Supplementary Figure 1) in breakout groups to promote equity and democratization in AI AgeTech design (Rubeis et al., 2022). Three personas were presented, illustrating the care ecosystems of older adults at different functional levels. Drawing on the diverse lived experiences of WG members, we “brought to life” our personas by specifying additional details and nuances with which we could empathize and relate. We used additional visual probes to generate scenarios in which personas used various smart homes (Figure 4) and assistive robot functions with different desired levels of automation. We used additional visual probes to explore lived experiences, feelings toward, and speculations about how intelligent wheelchairs may transform care or social interactions in care homes (Figure 5).

## Synthesizing Humanistic Attributes and Values to Guide AI AgeTech Innovation

**Figure 6.**
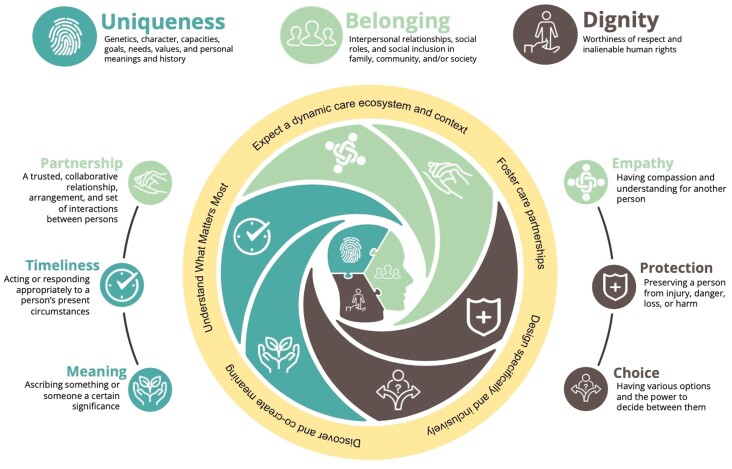
A guiding model for humanistic AI AgeTech innovation. Our guiding model is comprised of three humanistic attributes (silhouette, center), six humanistic values (middle circle, see also Table 1), and five design orientations (outer circle, see also Supplementary Table 1) to guide AI AgeTech innovators. AI = artificial intelligence.

We propose a preliminary guiding model for humanistic AI AgeTech innovation (Figure 6) that integrates theoretical concepts of humanism and our WG insights and synthesis. Drawing on conceptualizations of humanism from geriatrics, social robotics, and humanistic AI ethics, we use “humanistic” to underscore the vulnerability, uniqueness, and value we share as humans (Coghlan, 2022); emphasize *interpersonal relationships* involving the giving and receiving of care (Leibing, 2019); and connote the *pluralism and incommensurability of values*, whereby humans “are confronted with practical problems that implicate an array of values that pull in different directions” (Tasioulas, 2022, p. 236). Synthesizing these concepts with experiences shared in our WG, our model proposes three humanistic attributes, six interrelated humanistic values (Table 1) that reify and reinforce the attributes, and five design orientations intended to guide AI AgeTech innovators. The values are placed in a circle according to their relatedness to other values. Below, we elaborate on each attribute and value, relate them to published literature and ethical concepts, and cite examples from our WG discussions to illustrate how values may conflict, cooperate, or reinforce one another.

**Table 1.** Co-created Humanistic Values

Humanistic value	Lay definitions	Questions raised by working group
Protection	Preserving an actor(s) from injury, danger, loss, or harm	Who is (attempting to) protect whom, and from what? Do they want this protection? From what risks/harms are they being protected (e.g., physical, psychological, financial, social)? What are the costs or trade-offs of this protection?
Choice	Having various options and the power to decide between them	Who makes the final decision? Who influences the decision? Who is responsible for acting on the decision? Who is affected by the decision? Do actors have full transparency into how the system interprets/actions on their choices? What are actors’ personal values and beliefs?
Empathy	Having compassion and understanding for another actor	How can the AI intentionally communicate and respond to actors in ways that avoid emotionally harming or triggering them (e.g., with past trauma)? How can the AI detect emotional states for and between actors, and provide appropriate information and responses? How can the AI mirror empathy enacted in actors’ relationships with each other?
Partnership	A trusted, collaborative relationship, arrangement, and set of interactions between actors	How do the needs and/or values differ between actors? Where might there be opportunities for the AI to mediate or resolve value conflicts, or support aligned values, between actors? How can the AI mediate or enrich partnerships between actors? How can the AI, as an actor, improve the care ecosystem?
Meaning	Ascribing something or someone a certain significance	What meaning(s)—positive or negative—does each actor ascribe to the care arrangement? The AI? How might the AI foster positive meanings for and between actors? Have the actors or partnerships co-created new meanings through using the AI?
Timeliness	Actions or responses that are appropriate (for actors) in their present circumstances	Does the AI’s function fit with each actor’s present health, capacities, priorities, and circumstances? The care ecosystem’s needs at the present time? Have actors developed adequate experience, skills, and trust in the AI right now? Is the level of automation and intelligence appropriate for each actor and their care arrangements right now? Might individual actors have different needs/tolerances for support or automation at different times (e.g., day vs night; alone vs together)? How can the AI be modified to better respond to present needs, values, capacities and circumstances?

*Notes*: A set of humanistic values, co-created lay definitions, and questions discussed during working group sessions about “AI AgeTech.” To define terms, “AI” refers to artificial intelligence, in which the technology—to some extent—predicts, reasons, and automates action. “AgeTech” refers to technological products or services intended to support older adults and/or their care partners; and “AI AgeTech,” therefore, refers to AgeTech that is enabled by artificial intelligence. In the table, “actor” refers to a human actor (e.g., older adult, care partner) and “the AI” refers to an AI AgeTech (e.g., smart home, socially assistive robot, intelligent wheelchair). AI = artificial intelligence.

### Dignity: Balancing the Protection and Choices of an Older Adult

We define dignity as the worthiness of respect and rights to which every person is entitled, and which is actualized through the values of choice and protection. Notably, most previous ethical discussions related to AI AgeTech (e.g., on personal privacy, autonomy, data security, informed consent, loss of liberty, felt objectification, infantilization, deception) have focused on promoting dignity, protection, and choice for older adults—primarily, at the individual level.

To explore the interpersonal level, we disentangled the complexities of balancing the protection of older adults with respecting their choices. Protecting the physical safety of older adults tends to incur costs to their privacy, autonomy, or sense of dignity. A common relational conflict ensues when care partners/providers (attempt to) implement protective measures (e.g., hiring a care worker or installing monitoring technology) against an older adult’s choice. Although we assert that older adults have the right to take risks that affect themselves, respecting this right becomes especially complicated when a cognitive condition impedes self-awareness of deficits (i.e., anosognosia; Tannou, 2023). Care partners/providers may feel responsible for protecting the older adult but may be ineffective in doing so without their agreement.

Actors may reflect on their different values (Hechinger et al., 2022; Hwang et al., 2017) and attempt to reconcile conflicts. A care partner may resist implementing protective measures to avoid threatening an older adult’s dignity or causing them psychological harm (e.g., feeling stigma or insecurity). Older adults experiencing anxiety about protection (e.g., when alone overnight) may resist seeking protective measures from care partners to avoid burdening or giving them cause to consider a care home placement. Under such circumstances, determining what matters most to older adults (Moye et al., 2021) may facilitate compromise resulting in acceptance of a technological arrangement if they ascribe greater meaning to the outcome (i.e., staying in their homes; Zhu et al., 2022).

Adjacent values also influence protection and choice. When care partners enact empathy with the goals and meanings held by the older adult, a collaborative partnership that balances actors’ different agendas may be created. For example, an activity monitoring system may fulfill a care partner’s priority to know whether an older adult is safe at home, while facilitating communication (e.g., overcoming “phone tag”) valued by the older adult (Figure 4). Reciprocally, older adults enact empathy toward care partners when choosing to accept technological support that protects care partners from psychological or practical stress and affords them a sense of security. When mutual empathy is not established, implementing measures against an older adult’s choice risks psychologically harming them, harming relationships, and creating safety risks if the older adult conceals their functional challenges thereafter. Such situations may occur when older adults exhibit anosognosia and others resort to deceptive yet empathic ruses to convey respect for older adult’s choices while protecting them.

There may be important constraints to an AI demonstrating empathic actions that respect older adults’ choices while trying to protect them. If an older adult who is living alone craves food outside her dietary guidelines, her robot, if designed as a nonjudgmental companion, may comply with her request, respecting her right to take the health “risk.” A more complicated scenario may occur if a care home resident expresses loneliness and instructs the home’s robot to accompany her outside. Indeed, robots can communicate with empathy (e.g., validating feelings), but whether they comply raises questions as to whom (i.e., “owner”) and for what the robot is responsible (e.g., care home and its policies). It is, therefore, important that an AI’s responsibilities, accountabilities, and loyalties (Vallor, 2011) can be clearly specified in the design and learning processes and made transparent to users to mitigate social risks and conflicts of interest (Aguirre et al., 2020).

### Belonging Through Partnership and Empathy for All Actors in the Care Ecosystem

Belonging to social groups is fundamental to humanism. We view belonging as encompassing a person’s inclusion and participation in multiple social groups (e.g., family, community, sociocultural, ethnic, or religious group, society). Belonging is actualized by the values of partnership with others in one’s care ecosystem, and empathy shared in one’s interpersonal relationships. It is through interpersonal relationships that one feels a sense of belonging to, and identification with, social groups. Discussed concepts in biomedical ethics—beneficence, nonmaleficence, and desire for human contact—relate to our humanistic attribute of belonging.

Fostering partnership within a care ecosystem facilitates a sense of belonging for all actors, but requires adaptation with time and changing circumstances (Figure 1). Partnerships undergo change and evolution as actors, needs, values, and circumstances change (Hwang, 2019)—thus, partnership and timeliness are seen as mutually influencing values. Actors within interdependent care relationships foster partnership when they learn (or unlearn), adapt, and pivot toward better ways of caring for one another with time and experience (Dupuis et al., 2012; Hwang, 2019). A care worker, for example, may change how an older adult takes care of himself, as well as the routines of family care partners. When circumstances change (e.g., death of a care partner), roles and relationships within the ecosystem adapt to regain the sense of partnership.

An AI as a care ecosystem actor may mediate, complement, or augment partnership between and with human actors. A care robot adopted to support an older adult with everyday functioning may restore a social relationship quality between the older adult and an overwhelmed care partner. Figure 4 illustrates how collaboratively adopting an AI may facilitate shared caregiving and coordination between care partners; inform care partners about an older adult’s activities, moods, and health behaviors; and provide peace of mind and affirmation of their positive impact as care partners.

An AI, as a care actor, has the potential to bridge protection and partnership through its empathic capacities. In future developments, an AI may learn through observation of positive interactions between human actors, and mirror or adapt effective forms of communication. Alternatively, it may learn when relational interactions between human actors become negative or harmful and play a complementary role by offering a positive interaction (e.g., friendly companionship-like interaction, reassurance to distressed care partners). Advances in “affect-aware” AI AgeTech (i.e., AI that responds to emotional states of humans and interactions [Hoey et al., 2016]) may enable future systems to respond with compassion and nonmaleficence to actors’ idiosyncratic needs. Given the advanced social capabilities of certain AI AgeTech—such as care robots (Prescott & Robillard, 2021)—its abilities to sense and empathically respond to older adults’ emotions (e.g., when triggered by past trauma) may be more effective than some humans. This capability may be especially valuable for persons living with dementia, who rely largely on emotional experience (Guzmán-Vélez et al., 2014). Just as humans tend to reciprocate feelings in relationships, cumulative positive interactions with an AI may foster trust in and promote partnership with the AI, potentially to benefit the entire care ecosystem.

### Uniqueness of Care Ecosystems, Circumstances, and Meanings Ascribed to Care

We consider uniqueness to be a biopsychosocial feature of every human, where one’s genetic composition and experiences manifest in a distinctive physiology, personality, functional capacity, personal history, and set of values and goals. We note that previous literature has yet to discuss how AI AgeTech addresses human uniqueness, beyond ethical discussions that promote inclusive design based on using datasets and algorithms that mitigate algorithmic bias (Chu et al., 2021; Robillard et al., 2018).

We co-constructed uniqueness as an attribute extending beyond the individual to the care ecosystem, which will be unique at any point in time (Figure 1). Our value of timeliness emphasizes that AI AgeTech should respond to the temporal dynamics of often changing health and care circumstances (Hechinger et al., 2022; Hwang et al., 2017, 2020); care actors’ human relationships (Prescott & Robillard, 2021); their meaning-making with care experiences (Hwang, 2019); and their experiences exploring and using technologies to support care situations (Hechinger et al., 2022; Hwang et al., 2020). These dynamics influence actors’ technology acceptance and specific preferences related to AI automation. For instance, if an older adult is experiencing health changes that create time-sensitive demands on care partners, actors may not have interest or capacity to assimilate an AI actor into the care ecosystem. Actors, however, may be more willing to adopt the AI if practical care arrangements stabilize, or if the AI delivers a meaningful solution that mediates value conflicts (safety vs social connectedness, Figure 4). Another example may be a care home resident with early-stage dementia attempting to use an intelligent wheelchair. The unfamiliar semiautonomous driving may exacerbate their anxieties related to their cognitive decline and their socially unpredictable setting (Viswanathan et al., 2017). An AI should therefore “meet people where they are” and be able to learn and adapt as changes occur.

Temporal dynamics (timeliness) may also facilitate AI AgeTech adoption, as actors develop proficiency and trust and ascribe new meaning to technology. older adults and care partners often need the prerequisite skills and timely training and support to successfully “take off” when adopting technologies (Hedman et al., 2016; Hwang et al., 2020). An older adult who successfully integrates a remote-controlled smart door lock into his “existing arrangements” (Gómez, 2015) may be motivated to explore a different level of automation (e.g., event-triggered door locking), or alternative application using the now-familiar technology (e.g., activity reminder). For multiple actors, exploring or adapting a care robot together may scaffold and catalyze social interaction (Prescott & Robillard, 2021), or provide a meaningful shared activity that promotes empathy, efficacy, and enjoyment (Hwang et al., 2020). In a care home, the use of intelligent wheelchairs may transform a care home context, potentially reducing certain social interactions while creating other social opportunities (Figure 5).

As demonstrated, actors “[complete] design in use” (Carroll, 2004) and should be expected to adapt an AI while, in turn, adapting their behaviors and attitudes toward it. They may discover benefits that assuage initial concerns (e.g., privacy) or, as AI AgeTech becomes more ubiquitous, accept risks out of “digital resignation”—where they resign themselves to digital surveillance, feeling it is beyond their control (Draper & Turow, 2019). The interplay between timeliness and meaning can be influential to whether an AI AgeTech fits within a unique care ecosystem.

## Designing AI AgeTech for Dynamic Care Ecosystems

As shown in our model (Figure 6), we propose design orientations with practical considerations, tools, and techniques (Supplementary Table 1) for innovators who are designing, developing, and evaluating AI AgeTech to support older adults’ care ecosystems.

### Understand “What Matters Most” Right Now, and to Whom

We emphasize the importance of understanding what matters most to which actors at the present time. Employing the What Matters Most geriatric tool (Moye et al., 2021) can identify older adults’ values related to functioning (e.g., choosing where to live), enjoying life (e.g., participating in hobbies), and connecting socially (e.g., having good relationships). Using constructs from this tool with other care actors may identify how their values differ from the older adults in question, and where there may be conflicts and opportunities for AI AgeTech to mediate. This tool may help innovators to explicate their innovation’s goals, consider assumptions about target users, and guide market analysis. For instance, before making investments in a product that enhances functioning, innovators may survey how their target market prioritizes this vis-à-vis social connection or enjoyment. Personas (Supplementary Figure 1) and care maps (Figure 3) may offer complementary techniques to specify the care ecosystems to be targeted.

### Expect a Dynamic Care Ecosystem and Context

Elaborated in the value of timeliness, “What Matters Most” to different actors may change with time, circumstance, and care and technological experience. An AI AgeTech innovation designed for a care ecosystem at one point in time may need to adapt to these changes, otherwise risks becoming irrelevant. Innovators also need to understand the nature of the care context (e.g., older adult’s home, care home) and the constraints and policies that govern it. Caution must be exercised to constrain what an AI AgeTech *should* do in a context, from what it *can* do. AI parameters and constraints need to be specified in collaboration with actors in that context, as an AI’s full capability may be inappropriate or potentially harmful. In care home settings (Figure 5), for example, intelligent wheelchairs may increase residents’ freedom to socialize but may create new risks (e.g., increased staff demands) that require constraints or policies to be specified (e.g., residents may not enter others’ private rooms).

### Foster Empathic Care Partnerships Where AI May Be an Actor

Understanding “What Matters Most” to different care actors identifies conflicts and thereby opportunities to foster empathic partnership between actors, and potentially augment the care ecosystem with an AI actor (Rattay et al., 2023)—as a companion, mediator, or additional care resource. Given its unique capabilities, AI AgeTech is expected to change risks and relations between human actors (Vallès-Peris & Domènech, 2023). A smart home or assistive robot that interacts with different actors may be perceived by the older adult as more companion-like and less paternalistic than an “overprotective” care partner. Alternatively, this ability to respond (i.e., without overt instruction) to the older adult may introduce new problems that care partners feel responsible to manage. Another consideration is whether an AI should be “loyal” to one actor over another (Aguirre et al., 2020) or fulfill a mediating role, helping human actors resolve their value conflicts. Especially with “communicative AIs” (e.g., carebots) that offer social interaction, the way human actors interpret the AI’s role (Guzman & Lewis, 2020) may shift relationships within the care ecosystem.

### Discover and Co-create Meaning With Actors as They Use AI AgeTech

Accordingly, deploying the AI and iteratively designing with actors over time, adaptation, and experience are paramount. Engaging directly with older adults, in particular, may overcome biases and stereotypes (e.g., digital ageism). Longitudinal co-design engagements with care actors (including care providers [Chu et al., 2021]) also afford innovators opportunities to study and refine the AI, as “users” mutually adapt with it, ascribe it new meanings (e.g., trusted friend), or discover unexpected purposes. These insights may generate new parameters for user specification or machine learning. As users renegotiate their desired levels of control versus automation, allowing for “gradations of support” (Piper et al., 2016) as human capacities fluctuate and evolve, may promote human-human and human-AI care partnerships over the longer term. Co-designing with users over time allows for a more rigorous analysis of the ethical and social consequences of the AI, and guides technological adjustments that may mitigate potentially harmful scenarios and promote beneficial scenarios (Prescott & Robillard, 2021).

### Design for Uniqueness, Specifically and Inclusively

Harnessing the uniqueness of individuals and care ecosystems may promote inclusive design and equitable use of AI AgeTech. It may be possible to leverage AI to identify and learn the key parameters of differences between care ecosystems and generate meaningful profiles of ecosystems that share similar needs and usage patterns (e.g., ecosystems including persons with different cognitive diagnoses or collectivistic values). Learning and iterating from “edge cases” with the greatest heterogeneity of characteristics (Treviranus, 2019) may promote inclusive design by addressing unique needs that may resonate with others in similar circumstances.

Designing for unique and specific care ecosystems also promotes inclusion and equity. Algorithmic bias occurs when AI algorithms are specified and trained with biased datasets, which perpetuate technology-mediated cycles of injustice (e.g., discrimination, ageism) when the technology only responds to the stereotypical actors, care ecosystems, and contexts (Chu et al., 2021). If a smart home innovator, for instance, assumes that all older adults have care partners for technological support, then older adults without care partners may be excluded from both the initial design, algorithmic training, and learning processes that improve the system. If this innovation gains widespread traction, it will not support older adults without care partners. Although every care ecosystem is indeed unique and dynamic, AI-based strategies may help to identify relevant characteristics and algorithmic parameters such that machine learning responds to these differences. Hence, designing AI AgeTech specifically and inclusively not only mitigates bias and discrimination, but may enhance the accuracy of AI parameters to support a broad diversity of older adults, care partners, and actors in the ecosystem.

## Conclusion

Amidst growing ubiquity and investments into AI innovation, designing AI AgeTech *humanistically* not only serves ethical imperatives, it delivers what matters most to older adults and care partners, supporting their dynamic relationships and care experiences, and empowering them through inclusive design processes that foster technological literacy and collaboration—thus, optimizing social and economic resources. In this article, we advance previous ethical discussions with a humanistic approach to design that captures the complexity, fluidity, and interdependency of dynamic care ecosystems; the conflicting values between human actors; and considerations of how introducing an AI—as an actor—may transform these relationships. We offer a preliminary model of humanistic attributes, values, and corresponding design orientations that may guide AI AgeTech researchers and innovators in practice to promote successful aging in AI-enabled futures. Future research involving diverse care ecosystems is needed to validate and further develop this model.

## Supplementary Material

gnae093_suppl_Supplementary_Material

## Data Availability

As the authors formed a working group to generate, synthesize, and coauthor the ideas presented, there are no data to report. The requirements for data availability and preregistration are therefore not applicable.
