# Supplementary material for: Co-creating Humanistic AI AgeTech to Support Dynamic Care Ecosystems: A Preliminary Guiding Model
Source: Gerontologist. 2024 Aug 2;65(1):gnae093. doi: 10.1093/geront/gnae093 (PMC11648309; doi:10.1093/geront/gnae093)
Supplement: gnae093_suppl_Supplementary_Material [file gnae093_suppl_supplementary_material.docx]

**Supplementary Material**

**Supplementary Figure 1**

*Preliminary personas developed at working group sessions*


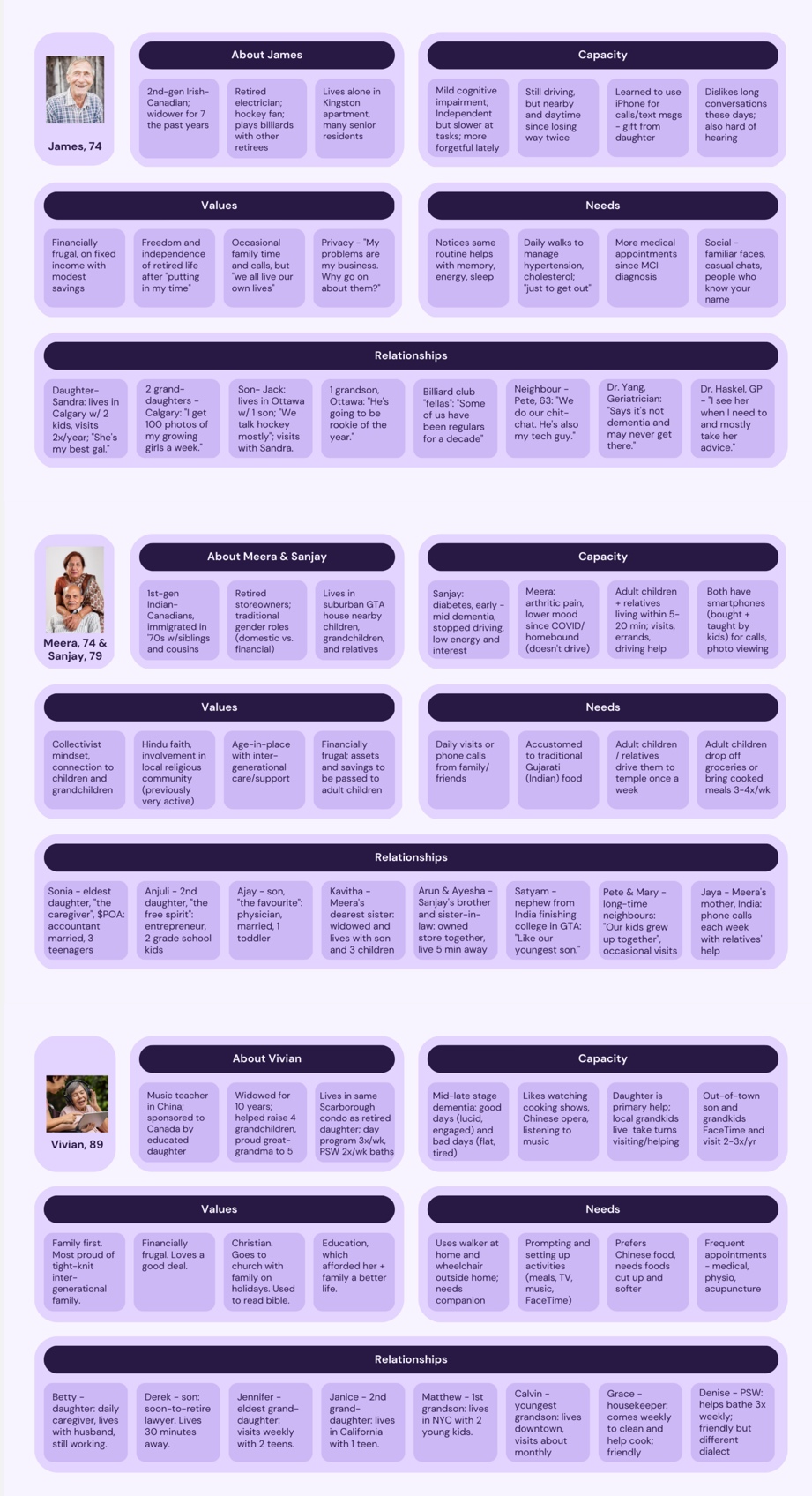


*Note.* These preliminary personas were developed by ASH and RHW, based on previous research (Hwang et al., 2017, 2019, 2020) and working group members’ expressed lived experiences, and shared at the second working group session (half-day, in-person) to guide breakout activities. Assigning one persona to each (of three) breakout groups, working group members (i.e., co-authors) “brought to life” their personas by adding details and discussing how their personas might respond to, use, and adapt to smart home technologies or socially assistive robots.

*Alt text*. Summarizes three personas – James, Meera and Sanjay, and Vivian – who are older adults with progressively more care needs. Each persona encompasses five sections: About the persona, their capacities, values, needs, and social relationships.

**Supplementary Table 1**

*Co-created humanistic values*

| Design orientation | Key questions to consider | Recommendations for implementation |
| --- | --- | --- |
| Understand “What Matters Most” to whom, and right now? | - How do values align and conflict between care actors (especially older adults and primary care partners)? - Might “What Matters Most” have changed recently, or might it change soon? | - Refer to the “What Matters Most” tool (Moye et al., 2022) when designing questions for different care actors to understand their values and value conflicts; - Develop personas (Adlin & Pruitt, 2010) of key actors (Supplementary Material, Figure 1) to build understanding and empathy with them, and to explicate any assumptions about their needs, values, etc.; - Update the above when there are changes to the care ecosystem (e.g., health/functioning, living/care arrangements, new AI usage). |
| Expect a dynamic care ecosystem and context | - Home context: - (How) does the AI accommodate for the less predictable context in which actor’s behaviours take place? - (How) does the AI integrate with actors’ values, needs, routines, and arrangements in their home contexts? - Care home context: - How might the intended AI functions or use by one resident impact other residents? Visitors? Staff? Volunteers? - What might be the social implications of using the AI to each of the above actors? - What are the care home’s policies and norms related to technology use, decision-making, and risk management? | - Together with personas, develop care maps (e.g., Figure 3) for the care ecosystem to describe its composition, and the nature (e.g., frequency, support type) and quality of relationships between actors; - Involve care actors who regularly participate in the context in the research and design process, especially when introducing and adapting the AI in the target context(s); - For care home contexts, analyze the model of care; leadership capacity; staff, regulated professional and union roles; workload; policies and regulations related to resident/staff safety, leadership capacity and roles within the home, shared care decision-making processes, access to technology/devices, and overall culture of the care home. - Apply analysis to specifying the AI’s learning parameters, constraints, responsibilities, and loyalties. |
| Foster empathic care partnerships that consider the AI as an actor | - Considering “What Matters Most” to each actor, what value conflicts exist in the care ecosystem between actors? - (How) might the AI mediate, complement, or augment the relationships or interactions between actors, either to reconcile value conflicts, or reinforce What Matters Most? - Are there opportunities for care actors (including the AI) to redistribute or rearrange care in ways that synergistically strengthen the entire care ecosystem? | - Analyze the “What Matters Most” tool (Moye et al., 2022), personas (Adlin & Pruitt, 2010), and care map (e.g., Figure 3) to identify value conflicts; - Delineate specific role(s) that the AI, as an actor, will occupy in the care ecosystem, and re-map (on care map) the care ecosystem including the AI, modifying how relationships are expected to change between human actors. |
| Discover and co-create meaning with actors as they use AI AgeTech | - Before deployment: - Under what (social) specific conditions are we expecting the AI to perform what functions? - What level of control (actor) versus automation (AI) is appropriate in different scenarios/use cases? Is it appropriate to actors’ capacities and values? - What are the AI’s learning parameters?      - After deployment: - How are the actors and the AI learning from and adapting with each other? - Has using the AI, or any present circumstances, changed actors’ capacities or values (e.g., actors’ functional capacities, technology learning, trust in the AI, actors’ relationships)? - Have actors discovered new or unexpected ways of using the AI? - Has using the AI created new risks to any actors that need to be mitigated? - Would different levels of control versus automation be appropriate? - How has use affected the overall care ecosystem? | - Specify key scenarios or use cases, including the contextual conditions and expected interactions with actors (e.g., Figure 4); - Longitudinally analyze how different actors are learning, using, and adapting to and with the AI using mixed data/methods (e.g., usage statistics, participant observations, interviews, case study); - Update personas (Adlin & Pruitt, 2010), scenarios/use cases, and care maps (e.g., Figure 3) based on actual usage experiences, reflecting any new control/automation settings; - As needed, specify new AI learning parameters, constraints, and levels of control versus automation, allowing for “gradations” (Piper et al., 2016) whereby actors can adjust control to changing circumstances. |
| Design for uniqueness, specifically and inclusively | - What parameters characterizing actors, behaviours, relationships, and interactions are being specified vs. assumed in the algorithms? - Can AI be employed for data mining to generate profiles of different care ecosystems, to promote inclusion and resonance to other ecosystems with common characteristics? - (How) can new parameters be specified post-deployment, based on actual usage, in order to mitigate bias or discrimination? - Are the algorithms learning from one case or all cases?      - Who has the power, responsibility, and/or skills/ability to make these algorithmic modifications? | - Define personas, care maps, and scenarios/use cases before deployment to make explicit assumptions or biases; - Specify the AI with heterogeneous and “edge case” personas (e.g., ages, capacities, care ecosystem composition, ethnocultural / socioeconomic characteristics, living/care arrangements) to promote inclusive design (Treviranus, 2019); - Leverage AI capabilities to generate meaningful profiles of different care ecosystems, to promote inclusion with efficiency; - Re-specify the AI based across users based on deployment insights. |

*Note.* Detailed design orientations, key questions to consider, and guidance for implementing our guiding model of humanistic AI AgeTech innovation (Figure 6). Intended for practical use and future evaluation by AI AgeTech researchers or innovators. Here, ‘actor’ refers to a human actor and ‘the AI’ refers to an AI AgeTech (e.g., smart home, socially assistive robot, intelligent wheelchair).
